# HNRNPA2B1 confers immune escape of non-small cell lung cancer through targeting lactate/ferroptosis

**DOI:** 10.1038/s41419-025-08232-5

**Published:** 2025-11-21

**Authors:** Ye Zhang, Yeye Chen, Jiaqi Zhang, Xin Du, Cheng Huang

**Affiliations:** Department of Thoracic Surgery, Peking Union Medical College Hospital, Peking Union Medical College and Chinese Academy of Medical Sciences, Beijing, 100730 China

**Keywords:** Immunoediting, Cancer microenvironment

## Abstract

Immune escape and ferroptosis resistance contribute to non-small cell lung cancer (NSCLC) carcinogenesis. This study aimed to investigate the role of N^6^-methyladenosine (m^6^A) reader heterogeneous nuclear ribonucleoprotein A2B1 (HNRNPA2B1) in NSCLC immune escape and ferroptosis resistance. HNRNPA2B1 expression was clinically elevated both in the NSCLC samples and single-cell transcriptome sequencing (scRNA-Seq). In vitro co-culture with activated CD8^+^ T cells, HNRNPA2B1 high-expression hampered the CD8^+^ T cell-mediated killing effect and accelerated the immune escape. Besides, the enforced HNRNPA2B1 high-expression alleviated the ferroptosis. Furthermore, HNRNPA2B1 targeted the LDHA mRNA m^6^A modified site to enhance LDHA mRNA stability and lactate accumulation, thereby assisted NSCLC cells to evade CD8^+^ T antitumor immunity and reduced IFN-γ secretion by CD8^+^ T. Furthermore, IFN-γ could stimulate ferroptosis of NSCLC cells. Taken together, these findings revealed an important role for HNRNPA2B1 on NSCLC immune escape and ferroptosis resistance, which provided novel insight into m^6^A modification in NSCLC.

## Introduction

Non-small cell lung cancer (NSCLC) is one of most common malignancies affecting more than one million patients annually worldwide [1, 2]. Currently, the effective treatment for early-stage NSCLC is surgical excision. However, for advanced stage NSCLC, the efficacy using chemotherapies, such as platinum and fluoropyrimidine, is still limited and insufficient. Thus, a more comprehensive cognition for NSCLC pathophysiological Features is very essential to identify effective diagnostic and therapeutic targets.

N^6^-methyladenosine (m^6^A) marks, prevalent in the mRNA of eukaryotes, are controlled by three distinct types of enzymes: methyltransferases, referred to as the “writers”; demethylases, referred to as the “erasers”; and m^6^A-specific binding proteins, referred to as the “readers”. So far, m^6^A readers have been recognized for their roles in recognizing m^6^A modification. Among these readers, heterogeneous nuclear ribonucleoprotein A2B1 (hnRNPA2B1) presents an important role across various cancers. It is implicated as a promoter of tumor growth in certain malignancies including NSCLC [3] and neuroblastoma [4].

Immunosuppression is closely related to the occurrence and development of tumors. Tumor immune editing always occurs when immune system recognizes tumor cells, resulting in the resistance to immunotherapy [5]. In tumor microenvironment, tumor cells evade the immune killing through series of ways, such as abnormal tumor surface antigens expression, tumor cells surface molecular structure variation [6]. Tumor cells could also secrete immunosuppressive factors to inhibit tumor immune response.

Ferroptosis is a novel cell death known as iron death. The disorder of iron homeostasis is related to a variety of tumors, such as apoptosis, chemotherapy resistance and so on. Clinically, elevated serum iron level in tumor is always associated with cancer grade. In NSCLC tumorigenesis, the oncogenes promote NSCLC progression by acting as a suppressor of ferroptosis, independent of SLC7A11, GPX4 or NRF2 [7]. Besides, the ferroptosis could be induced by CD8^+^ T cells to kill nearby cancer cells on IFN-γ production [8]. Immune checkpoint blockade could trigger tumor ferroptosis, suggesting the interaction within immune escape and ferroptosis resistance [9].

Here, this study focused on the roles of m^6^A reader heterogeneous nuclear ribonucleoprotein A2B1 (HNRNPA2B1) on NSCLC ferroptosis and immune escape. Findings demonstrate that HNRNPA2B1 triggered LDHA/Lactate pathway in NSCLC cells, resulting in the repression of CD8^+^ T cells mediated antitumor. In-depth mechanisms also found that IFN-γ secreted by CD8^+^ T stimulated ferroptosis of NSCLC cells. These findings showed an important role for HNRNPA2B1 on NSCLC progression and provided novel insight into m^6^A modification in NSCLC ferroptosis and immune escape.

## Materials and methods

### Clinical samples

For the study, individuals diagnosed with non-small cell lung cancer (NSCLC) and receiving treatment at Peking Union Medical College Hospital were selected as participants. Samples of both tumor and surrounding non-cancerous tissues were obtained at the time of surgery, with some specimens being preserved in paraffin blocks and others flash-frozen in liquid nitrogen for subsequent analysis. All participants provided written informed consent prior to inclusion. Specimens were gathered from participants, with informed consent secured from each individual, following the approval by the Peking Union Medical College Hospital’s Ethical Review Board in accordance with the Declaration of Helsinki.

### Cell culture and treatment

NSCLC cells (Calu-3, CVCL_0609; SK-MES-1, CVCL_0630; A549, CVCL_0023; H1299, CVCL_0060) and control cells (human normal bronchial epithelial cell, 16HBE, CVCL_0112) were purchased from ATCC and maintained in RPMI-1640 medium that supplied with 10% FBS (BI, Israel) and 1% penicillin and streptomycin (Sigma, USA) and maintained in 37 °C incubator with 5% CO_2_. Cells were seeded in culture plates and incubated overnight. For iron-induced death, cells were then treated with erastin. Ferroptosis inhibitor Fer-1 (10 μM) was performed to reduce the iron-induced death. IFN-γ (cat. HY-P7025) was utilized to treat NSCLC cells (500 IU/mL).

### Plasmids construction and transfection

For HNRNPA2B1 knockdown, shRNA lentiviruses and corresponding control were synthesized by GeneChem (Shanghai, China) and infected into cells. Then, the cells were selected using puromycin (Biosharp, 2 μg/ml). To construct a plasmid overexpressing HNRNPA2B1, the coding sequence of human HNRNPA2B1 was synthesized and cloned into vector (GV492, GeneChem). The lentivirus containing Flag-tagged HNRNPA2B1 gene were transfected. For knockdown of LDHA, siRNAs targeting LDHA were purchased from the GenePharma (Shanghai, China) and transfected into cells with Lipofectamine 2000 (Invitrogen).

### RNA isolation and real-time quantitative PCR

Total RNA was isolated from each samples using TRIzol reagent for reverse transcription. The complementary DNA (cDNA) was generated using SuperScript First-Stand Synthesis system (Invitrogen) according to the manufacturer’s instructions. The qPCR was conducted using PCR Master Mix (Applied Biosystems) on an ABI7500 Real-time PCR System using primers sequences listed in Table S1.

### T cell activation and co-culture system

Peripheral blood samples were obtained from donors and the peripheral blood mononuclear cells (PBMCs) were purified. For the activation T cells, PBMCs were incubated by Dynabeads Human T-Activator CD3/CD28 (Gibco, USA) following the manufacturer’s protocols. In coculture of activated T cells and NSCLC cells, two types of cells were respectively placed in upper/lower chambers of Transwell (ratio of 10:1).

### Enzyme linked immunosorbent assay (ELISA)

NSCLC cells’ culture supernatant was collected and analyzed for cytokines production using ELISAs according to the manufacturer’s instructions. ELISA kit for human IFN-γ, TNF-α, Granzyme-B and Perforin.

### Flow cytometer analysis

For the surface PD-L1 expression, the PE-conjugated PD-L1 antibody (BioLegend, 1:200 dilution) was incubated in dark at 4 °C for 30 min following manufacturer’s introduction. Cells were tested in FACS Aria II Cell Sorter (BD Biosciences, CA, USA), and analyzed with FlowJo software (TreeStar).

### Cell proliferation viability analysis

The viability of NSCLC cells was measured by cell counting kit-8 (CCK-8, SolarBio, China) in line with the manufacturer’s protocol. NSCLC cells (5000 cells per well) were seeded into 96-well plate and incubated at 37 °C. At indicated time points, the CCK-8 reagent was added and incubated for 2 h. Then, the absorbance value at 450 nm were detected by microplate detector. For the colony formation, ten percent FBS was added to 1640 medium, which was used to maintain the transfected glioma cells in a brand-new six-well plate. A 14-day period was allowed for cell fixation using methanol and crystal violet staining (0.1%). We manually tallied the colonies that were visible.

### Cytotoxicity analysis

The T-cell mediated cytotoxicity on NSCLC cells was determined by LDH assay. After coculture of CD8^+^ T cells and NSCLC cells for 7 h, the culture supernatants were collected. The release of lactate dehydrogenase (LDH) into the supernatants was measured as an indicator of cell lysis using the Cytotoxicity Detection Kit PLUS (Sigma-Aldrich, catalog no. 04744926001) according to the manufacturer’s instructions.

### RNA immunoprecipitation (RIP) assays

To assess the binding of HNRNPA2B1 to LDHA mRNA, the EZ-Magna RIP Kit (from Millipore, USA) were performed according to instructions referring to previous reports [10, 11]. Initially, magnetic beads were coated with an anti-HNRNPA2B1 antibody (5 μg) and rotated overnight at 4 °C. The following day, these bead-antibody complexes were combined with total RNA for a 6-hour incubation at 4 °C. Subsequently, RNA was eluted, purified, and the relative interaction between HNRNPA2B1 to LDHA, namely the quantification of LDHA mRNA, was quantified using qPCR.

### RNA stability assays

NSCLC cells were cultured in 6-well trays and exposed to actinomycin D (5 μg/mL, provided by Sigma-Aldrich, USA) at intervals of 3, 6, and 9 h. In contrast, the control plates were treated with DMSO (Sangon, Shanghai, China) at the initial time point (0 h). Following this, total RNA was extracted through a method involving phenol, chloroform, and ethanol, which was then subjected to quantitative PCR analysis. The half-life of the mRNA was estimated using a linear regression model.

### Iron concentration and ROS analysis

After treatment, the iron load assay (Fe^2+^) of NSCLC cells was performed using the iron colorimetric assay kit (Applygen, Cat. E1042) referring to the manufacturer’s instructions. The lipid peroxidation was analyzed by Flow cytometry. The intracellular ROS was tested by DCFH-DA (Sigma-Aldrich, D6883) using confocal scanning microscopy.

### Xenograft model

Male C57BL/6 mice (10 mice, male) that aged 4 ~ 6 week-old were provided by Vital River Laboratory (China). Mice were randomly divided into two groups with free access to food and water. Mouse NSCLC cells (LLC) transfected with control or hnRNPA2B1 silencing vectors (5×10^6^ cells per 0.1 mL) were suspended in saline and inoculated into the flank. Tumor size was detected and calculated every three days. After feeding for 25 days, the tumors were excised and collected for weight. The IFN-γ level in tissue was detected by Mouse IFN gamma ELISA Kit (Cat. ab282874, Abcam). The Fe^2+^ level was tested by Iron Colorimetric Assay Kit (Elabscience, Cat. E-BC-K139-S). The care and use of laboratory animals in this study followed the National Institutes of Health guidelines. The experimental protocols were reviewed and approved by the Institutional Animal Care and Use Committee (IACUC), which operates under the oversight of the Ethical Committee of Peking Union Medical College Hospital.

### Statistical analysis

Data was calculated using SPSS 20.0 (SPSS, IL, USA) and plotted by Graphpad Prism 8.0 (GraphPad Software, CA, USA). Two-tailed student’s t test and ANOVA were performed to determine the statistical significance of the data. When *p*-value < 0.05, the difference was considered as statistical significance. All data were presented as mean ± standard deviation (SD).

## Results

### HNRNPA2B1 was upregulated in NSCLC

**Fig. 1:**
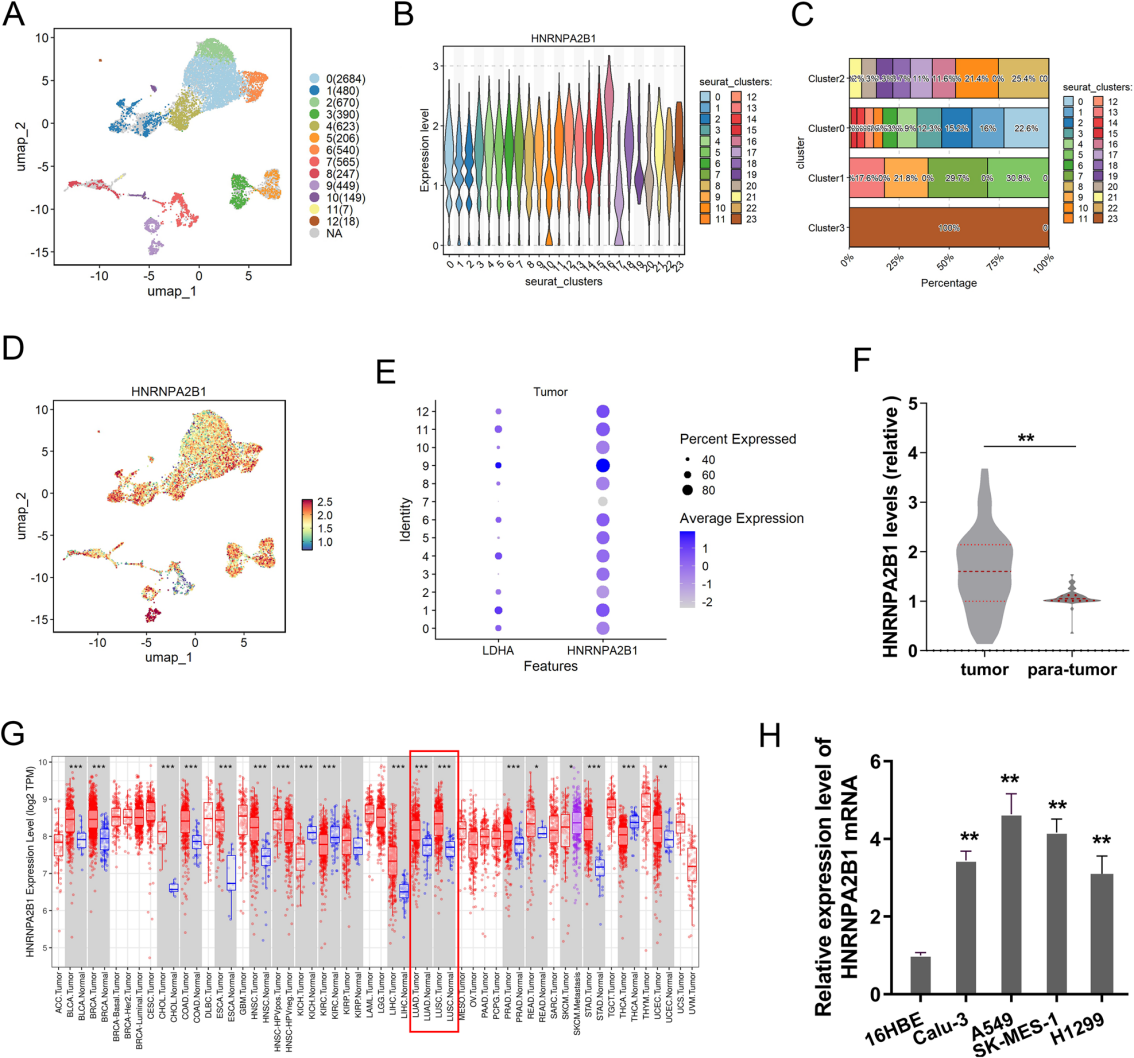
HNRNPA2B1 was upregulated in NSCLC. **A** Single-cell transcriptome sequencing (scRNA-Seq) in the NSCLC samples. **B, C** Expression of HNRNPA2B1 in scRNA-seq of NSCLC samples. **D** UMAP plot showed the distribution of HNRNPA2B1 in NSCLC samples. **E** The HNRNPA2B1 and LDHA level in NSCLC samples tissues. **F** The HNRNPA2B1 level in NSCLC clinical samples and para-tumor samples. **G** TIMER database reflected the level of HNRNPA2B1 in lung cancer (LUAD and LUSC). **H** RT-PCR showed the HNRNPA2B1 mRNA in the NSCLC cells (A549, PC-9, SK-MES-1, H1299) and normal cells (16HBE). *p < 0.05; **p < 0.01.

In the initial investigation, the role of HNRNPA2B1 had been verified in several human cancers [12]. Here, our research performed the single-cell transcriptome sequencing (scRNA-Seq) in the NSCLC samples (Fig. 1A). By observing the level of HNRNPA2B1 in several cellular subtypes, the data revealed that the level of HNRNPA2B1 in NSCLC tissue was higher in tumor cells than that in normal tissue (Fig. 1B, C). UMAP plot showed the distribution of HNRNPA2B1 in NSCLC samples (Fig. 1D). The expression of HNRNPA2B1 in NSCLC samples tissues was compared, as well as LDHA (Fig. 1E). Some samples were regarded as the high expression HNRNPA2B1 groups. In the NSCLC clinical samples, the HNRNPA2B1 levels also increased as comparing to the para-tumor samples (Fig. 1F). Lastly, the TIMER database reflected the level of HNRNPA2B1 in lung cancer. Results indicated that HNRNPA2B1 expression levels were higher in LUAD and LUSC (Fig. 1G). As respected, the HNRNPA2B1 level was highly expressed in the NSCLC cells (Fig. 1H). In conclusion, these data suggested that oncogenic HNRNPA2B1 was upregulated in NSCLC and positively correlated to PD-L1.

### HNRNPA2B1 repressed CD8^+^ T cells’ antitumor response to boost immune escape

**Fig. 2:**
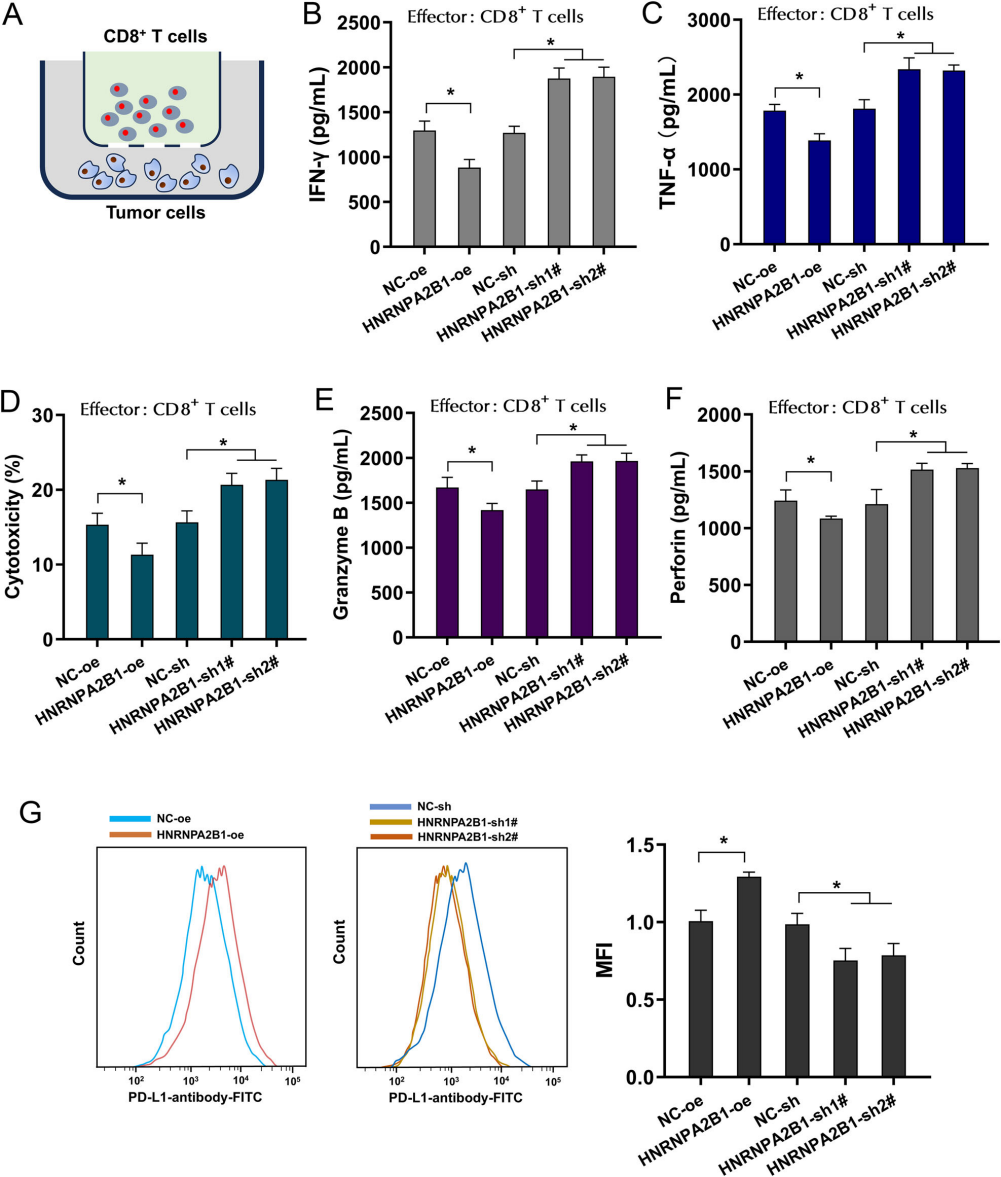
HNRNPA2B1 repressed CD8^+^ T cells’ antitumor response to boost immune escape. **A** The co-culture system was built using NSCLC cells (A549) and activated CD8^+^ T cells. **B, C, E, F** The secretion levels of cytokines by CD8^+^ T cells were measured by ELISA kit, including **B** IFN-γ, **C** TNF-α, **E** TNF-α, **F** Granzyme-B. A549 cells were respectively transfected with HNRNPA2B1 overexpression (HNRNPA2B1-oe) and silencing (HNRNPA2B1-sh1#, sh2#). **D** The cytotoxicity was measured by LDH release assays after incubation with activated CD8^+^ T cells. **G** The cell membrane PD-L1 in NSCLC cells (A549) with HNRNPA2B1 overexpression/silencing was analyzed by Flow cytometry using PD-L1 antibody. *p < 0.05.

Given the tight interaction within HNRNPA2B1 and immune infiltrating cells enrichment, the co-culture system was built using NSCLC cells (A549) and activated CD8^+^ T cells to investigate the role of HNRNPA2B1 on CD8^+^ T cells’ antitumor response (Fig. 2A). In coculture, the secretion levels of cytokines by CD8^+^ T cells were measured, encompassing IFN-γ and TNF-α. Results showed that CD8^+^ T cells secreted lower level of IFN-γ (Fig. 2B), TNF-α (Fig. 2C), TNF-α (Fig. 2E), Granzyme-B (Fig. 2F) when co-cultured with NSCLC cells transfected by HNRNPA2B1 overexpression. After incubation with activated CD8^+^ T cells, the cytotoxicity was measured by LDH release assays. The results demonstrated that CD8^+^ T cells executed lower cytotoxicity activity when co-cultured with NSCLC cells transfected by HNRNPA2B1 overexpression (Fig. 2D). While, the HNRNPA2B1 silencing exerted the opposite functions. To analyze the functional PD-L1, flow cytometer analysis revealed that HNRNPA2B1 overexpression promoted the functional PD-L1 expression, and HNRNPA2B1 silencing reduced the functional PD-L1 (Fig. 2G). In summary, these data suggested that HNRNPA2B1 repressed CD8^+^ T cells’ antitumor response to boost immune escape.

### HNRNPA2B1 repressed the ferroptosis of NSCLC cells

**Fig. 3:**
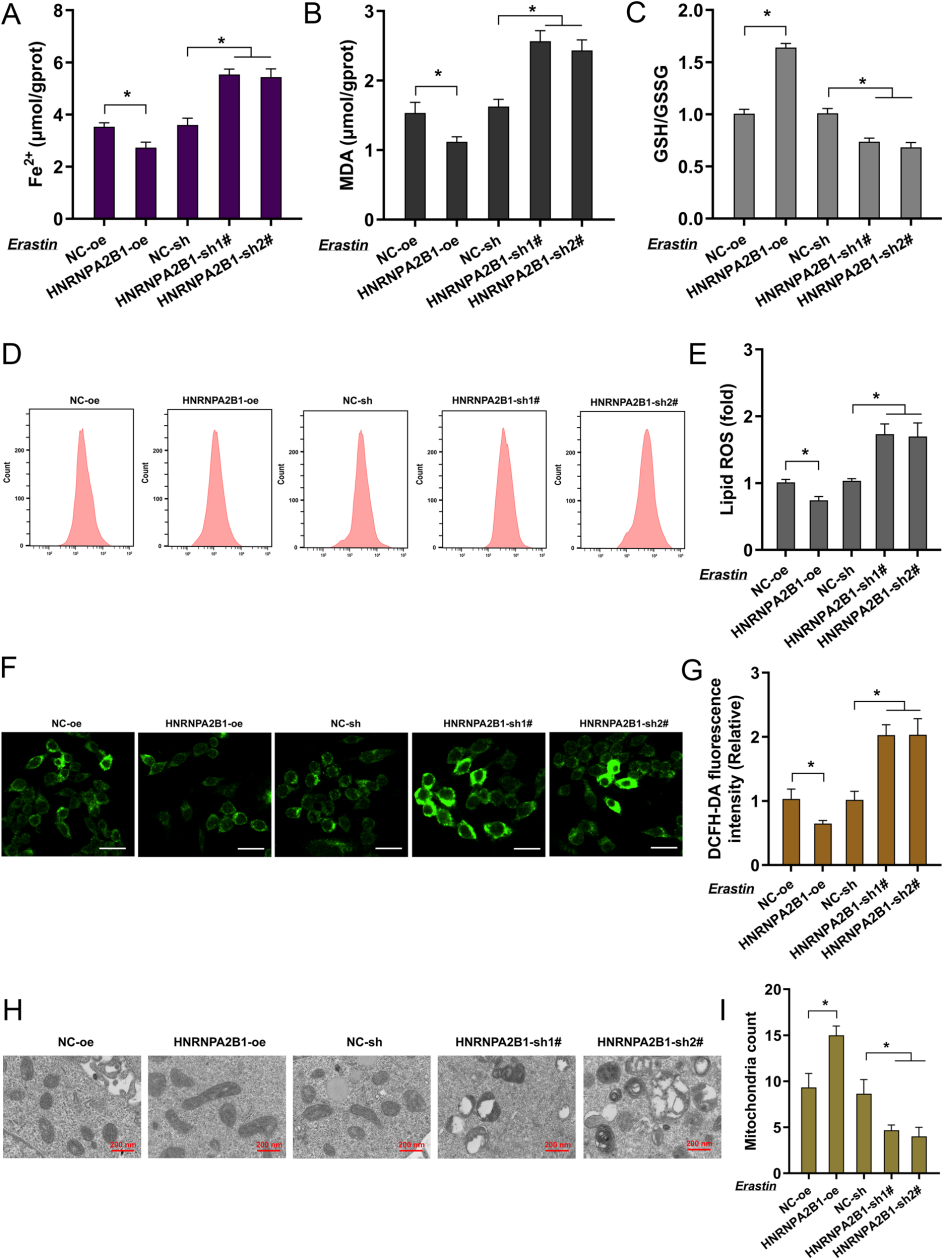
HNRNPA2B1 reduced the ferroptosis of NSCLC cells. **A** Fe^2+^ concentration, **B** Malondialdehyde (MDA), **C** Cellular glutathione (GSH)/oxidized GSH (GSSG) ratio were analyzed by kit in NSCLC cells with erastin administration (10 μmol/L). NSCLC cells were transfected by HNRNPA2B1 overexpression (HNRNPA2B1-oe) and silencing (HNRNPA2B1-sh1#, sh2#). **D, E** The lipid peroxidation was analyzed by Flow cytometry. **F** Representative DCFH-DA staining images. bar = 10 μm. **G** The quantitative analysis of fluorescence intensity. **H, I** The microscopic subcellular structures were observed by transmission electron microscope and quantified for mitochondria count. *p < 0.05.

Ferroptosis is a cell death mechanism induced by the accumulation of iron-dependent lipid reactive oxygen species (lipid-ROS), which ultimately leads to irreversible lethal damage to the cell. This process can be distinctly divided into two phases: the upstream phase involves the generation of ROS, while the downstream phase pertains to the actual execution of ferroptosis. In further investigation, our study focused on the interaction of antitumor response and ferroptosis and explored the underlying mechanism. Results indicated that, in the erastin treatment, the HNRNPA2B1 overexpression repressed Fe^2+^ (Fig. 3A), MDA (Fig. 3B), and lipid ROS (Fig. 3D, E), DCFH-DA intracellular ROS (Fig. 3F, G) accumulation and accelerated the GSH production (Fig. 3C), and alleviated the mitochondrial injury (Fig. 3H, I). Besides, HNRNPA2B1 silencing promoted the Fe^2+^, MDA, and lipid ROS accumulation, and reduced the GSH production, and aggravated the mitochondrial injury. From this, these data inferred that HNRNPA2B1 repressed the ferroptosis of NSCLC cells.

### HNRNPA2B1 recognized the m^6^A sites of LDHA to enhance its stability

**Fig. 4:**
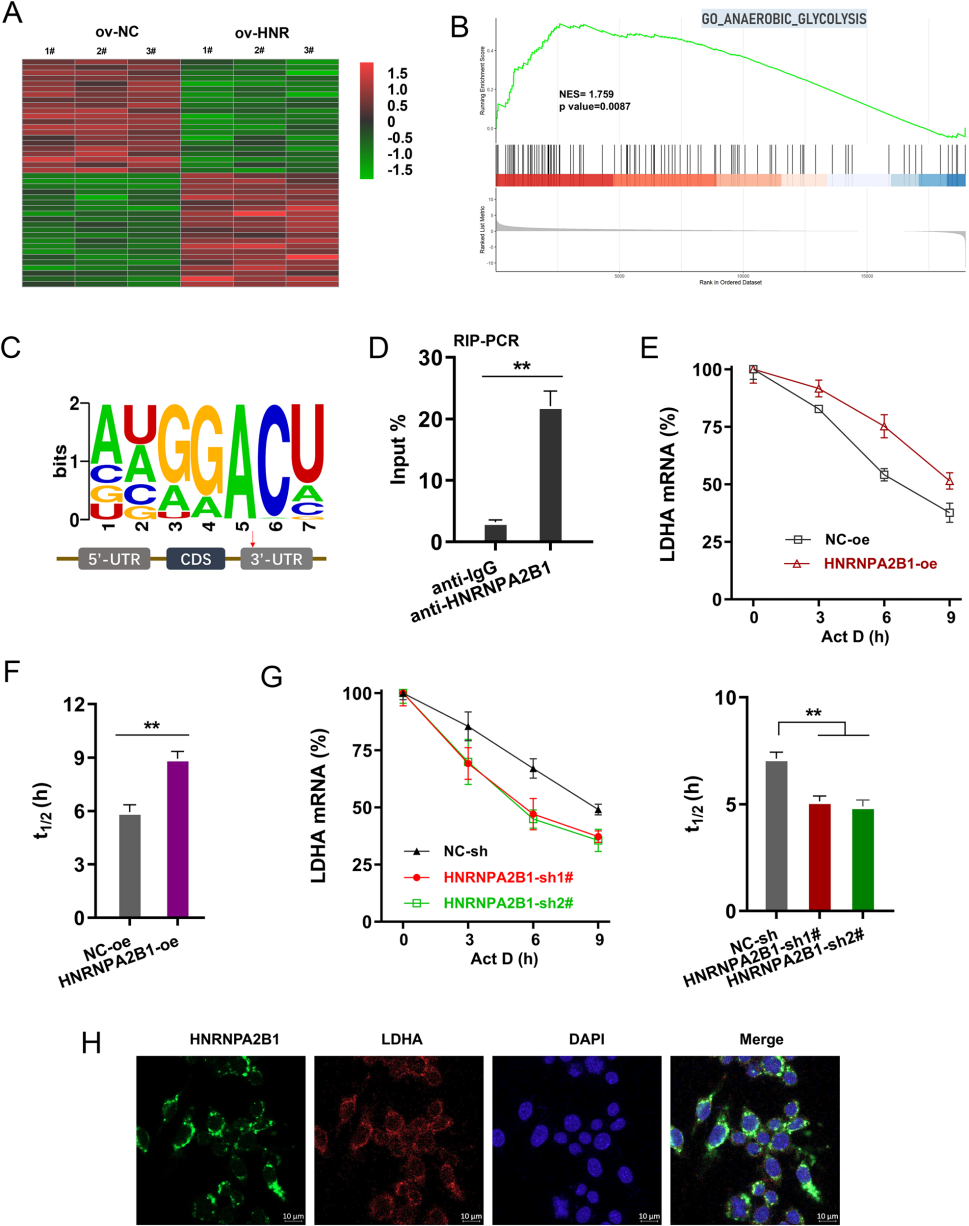
HNRNPA2B1 recognized the m^6^A sites of LDHA to enhance its stability. **A** Heatmap of RNA-seq data in A549 cells with HNRNPA2B1 overexpression and control. **B** GSEA analysis in HNRNPA2B1 overexpression cells compared with control cells in A549 cells. **C** Predictive analysis (SRAMP tool) offered potential m^6^A modified sites on the LDHA gene. The m^6^A modified motif was GGAC. **D** RIP-PCR assay was performed using HNRNPA2B1 antibody. The immunoprecipitated LDHA RNA was detected using PCR normalized to Input. **E–G** The stability assay by RNA decay analysis showed the LDHA mRNA half life time (t_1/2_) in NSCLC cells transfected with HNRNPA2B1 overexpression and silencing. **H** Immunofluorescence of HNRNPA2B1 and LDHA in the cytoplasm. *p < 0.05; **p < 0.01.

The RNA-seq data revealed that LDHA expression was increased upon HNRNPA2B1 overexpression (Fig. 4A). GSEA shows that ANAEROBIC_GLYCOLYSIS was altered in HNRNPA2B1 overexpression cells compared with control cells in A549 cells (Fig. 4B). Predictive analysis offered an interesting suggestion that there are several m^6^A modified sites on the LDHA gene. Besides, the m^6^A modified motif was GGAC (Fig. 4C). In order to identify whether HNRNPA2B1 bound with LDHA in NSCLC cells, the RIP-PCR assay was performed and results illustrated that HNRNPA2B1 remarkably bound with LDHA (Fig. 4D). For the LDHA mRNA fate analysis, the stability assay showed that HNRNPA2B1 overexpression up-regulated the LDHA mRNA stability (t_1/2_) (Fig. 4E, F) and HNRNPA2B1 silencing reduced the LDHA mRNA stability (t_1/2_) (Fig. 4G). HNRNPA2B1 and LDHA were both located in the cytoplasm (Fig. 4H). Collectively, the data from this part study revealed HNRNPA2B1 recognized the m^6^A sites of LDHA to enhance its stability.

### IFN-γ secreted by CD8^+^ T stimulated the ferroptosis of NSCLC cells

**Fig. 5:**
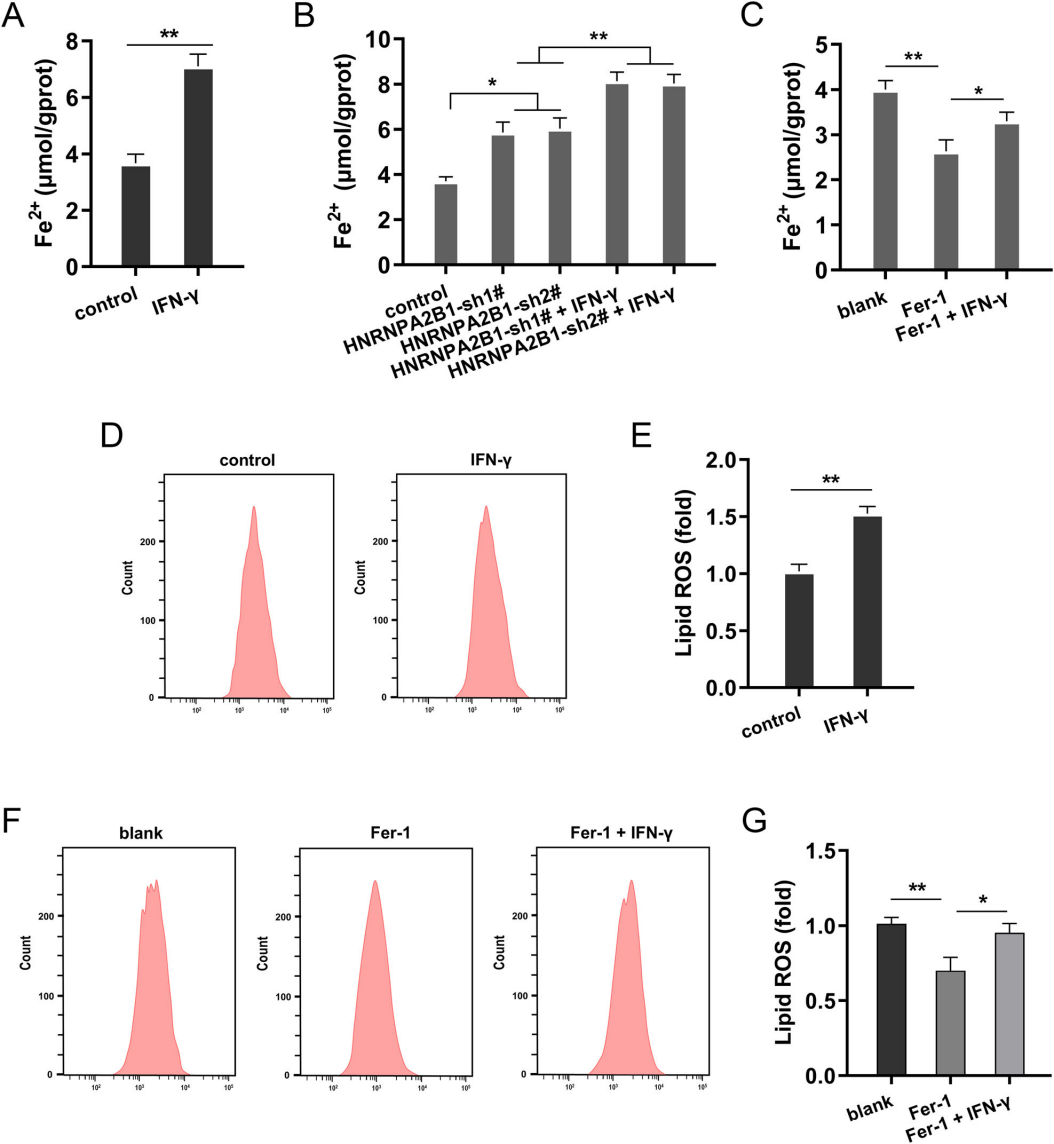
IFN-γ secreted by CD8^+^ T stimulated ferroptosis of NSCLC cells. **A** The iron concentration (Fe^2+^) accumulation was detected in NSCLC cells (A549). **B** The iron concentration accumulation was detected in NSCLC cells with HNRNPA2B1 silencing and IFN-γ (500 IU/mL). **C** The iron concentration accumulation was detected in NSCLC cells with Fer-1 (10 μM) and IFN-γ (500 IU/mL). **D–G** The lipid peroxidation was analyzed by Flow cytometry. *p < 0.05; **p < 0.01.

Given that HNRNPA2B1 could reduce the ferroptosis of NSCLC cells, the following assays were performed to investigate whether CD8^+^ T-mediated antitumor response regulated the ferroptosis of NSCLC cells. IFN-γ is a major cytokine secreted by CD8^+^ T that kills tumor cells. Firstly, the IFN-γ was administrated to NSCLC cells and results indicated that IFN-γ up-regulated the iron (Fe^2+^) concentration accumulation (Fig. 5A). Moreover, IFN-γ administration additionally up-regulated the iron (Fe^2+^) concentration accumulation in contrast with HNRNPA2B1 silencing (Fig. 5B). To test whether IFN-γ was activated in the administration of ferroptosis of NSCLC cells, the ferroptosis specific inhibitor ferrostatin-1 (Fer-1) was utilized to treated NSCLC cells. Results indicated that Fer-1 reduced the iron concentration accumulation, and IFN-γ co-administration restored iron level (Fig. 5C). Besides, the administration of IFN-γ promoted the lipid ROS accumulation (Fig. 5D, E). Meanwhile, Fer-1 reduced the lipid ROS accumulation and IFN-γ co-administration restored lipid ROS accumulation (Fig. 5F, G). Conclusively, the results affirmed that IFN-γ secreted by CD8^+^ T stimulated ferroptosis of NSCLC cells.

### HNRNPA2B1 targeted LDHA/lactate to represses CD8^+^ T cells mediated antitumor response and ferroptosis

**Fig. 6:**
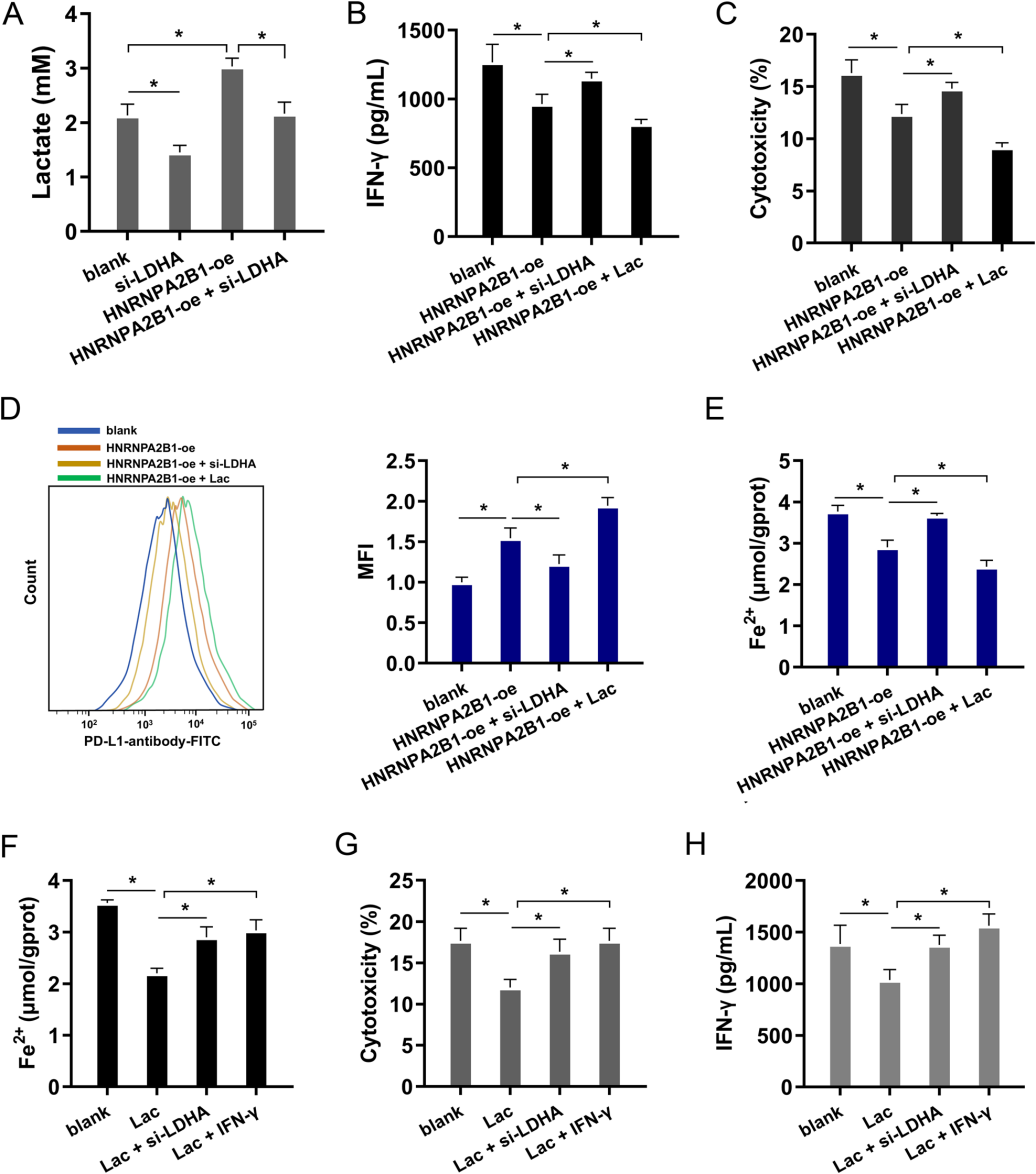
HNRNPA2B1 targeted LDHA/Lactate to represses CD8^+^ T/IFN-γ mediated antitumor response and ferroptosis. **A** The lactate concentration in co-culture supernatant. **B** The IFN-γ secretion levels of cytokines by CD8^+^ T cells were measured by ELISA kit. A549 cells were co-cultured with activated CD8^+^ T cells and respectively transfected with HNRNPA2B1 overexpression (HNRNPA2B1-oe), LDHA silencing (si-LDHA) or lactate (Lac). **C** The cytotoxicity was measured by LDH release assays after incubation with activated CD8^+^ T cells. **D** The cell membrane localized PD-L1 in NSCLC cells (A549) with HNRNPA2B1 overexpression, LDHA silencing or lactate (Lac) was analyzed by Flow cytometry using PD-L1 antibody. **E, F** The iron content (Fe^2+^) was tested NSCLC cells (A549) were treated with HNRNPA2B1-oe, LDHA silencing (si-LDHA), IFN-γ (500 IU/mL) and lactate (Lac). **G** The cytotoxicity and (**H**) IFN-γ were tested NSCLC cells (A549) were treated with HNRNPA2B1-oe, LDHA silencing (si-LDHA), IFN-γ (500 IU/mL) and lactate (Lac). *p < 0.05.

LDHA (lactate dehydrogenase A) accelerates lactate production by promoting aerobic glycolysis (Warburg effect) during tumor development and maintains the energy supply in anoxic environment [13]. Lactate accumulation mediated by LDHA can shape an immunosuppressant microenvironment and help tumors evade immune surveillance [14, 15]. LDHA plays a key role in maintaining intracellular pH homeostasis via lactate generation [16]. LDHA silencing repressed the lactate concentration in co-culture supernatant, and HNRNPA2B1 overexpression up-regulated it (Fig. 6A). In the rescue assay, HNRNPA2B1 overexpression transfection repressed the IFN-γ level (secreted by co-cultured CD8^+^ T) (Fig. 6B) and cytotoxicity (executed by co-cultured CD8^+^ T) (Fig. 6C), and accelerated the PD-L1 surface level (Fig. 6D). LDHA silencing co-transfection up-regulated IFN-γ level and cytotoxicity, and inhibited the PD-L1 surface level. Moreover, the lactate treatment could down-regulate IFN-γ level and cytotoxicity, and accelerated the PD-L1 surface level. For the Fe^2+^ concentration, the LDHA silencing and IFN-γ both increased the Fe^2+^ level, and lactate treatment reduced the Fe^2+^ level (Fig. 6E, F). For the CD8^+^ T cells mediated antitumor response, the cytotoxicity and IFN-γ were tested. Results indicated that the LDHA silencing and IFN-γ both increased their level, and lactate treatment reduced the levels (Fig. 6G, H). To sum up, the evidence supported the conclusion that HNRNPA2B1 targeted LDHA/Lactate to represses CD8^+^ T/IFN-γ mediated antitumor response and ferroptosis.

### HNRNPA2B1 silencing reduced the tumor growth, and accelerated the IFN-γ and Fe^2+^ level in vivo

**Fig. 7:**
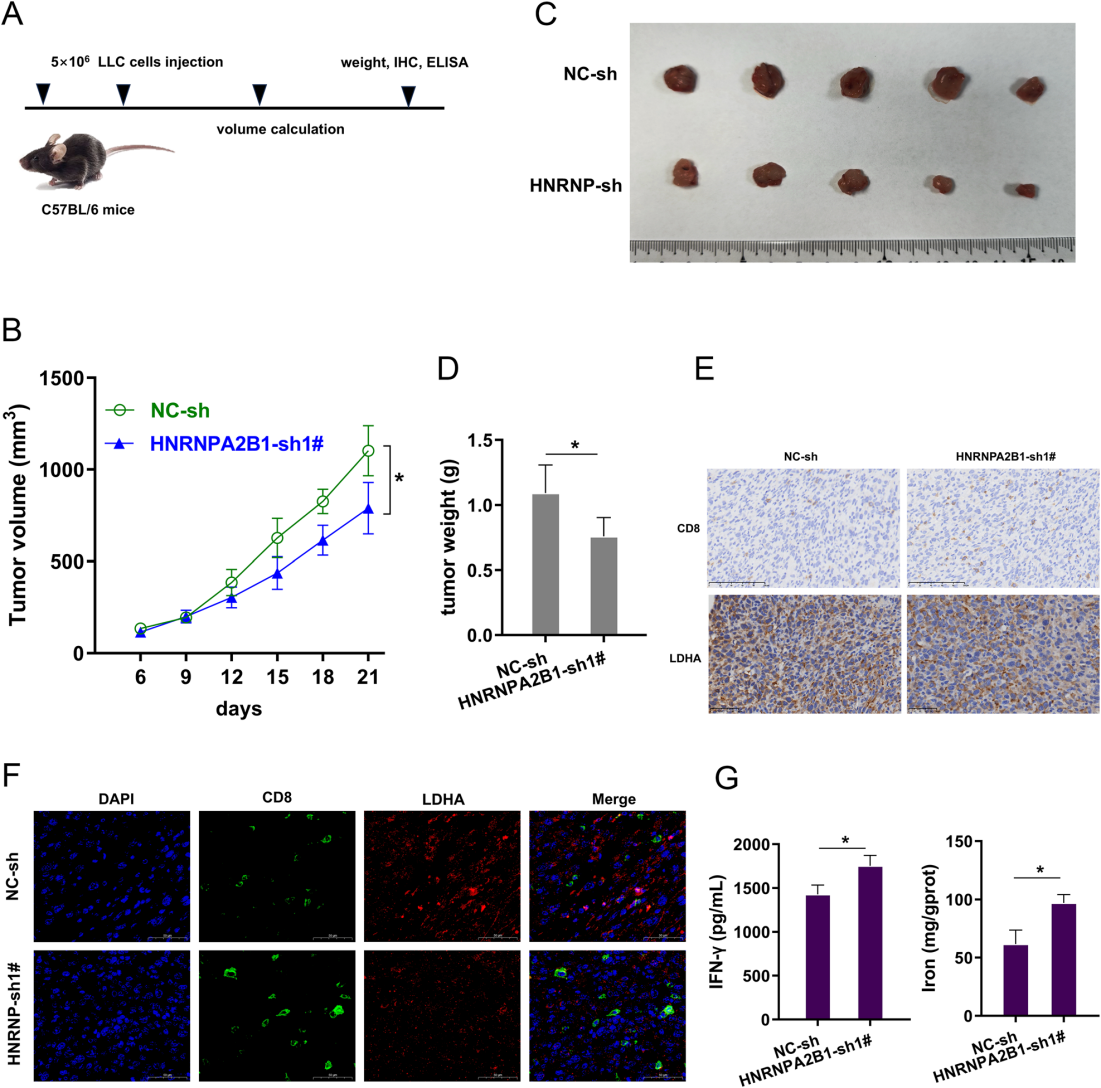
HNRNPA2B1 silencing reduced the tumor growth, and accelerated the IFN-γ and iron level in vivo. **A** The in vivo assay using C57BL/6 mice was performed. The mouse NSCLC cells (LLC) were transfected with HNRNPA2B1 silencing (sh-1#) or control (sh-NC), and then injected into mice. **B–D** The tumor growth was tested using NSCLC cells with HNRNPA2B1 silencing. **E** The immumohistochemical (IHC) staining of LDHA and CD8 positive cells. **F** Representative immunofluorescence (IF) images of CD8 and LDHA in HNRNPA2B1 silencing (sh-1#) or control (sh-NC) treated mice tissues. Scale bars: 50 µm at 400× magnification. **G** The IFN-γ and iron (Fe^2+^) concentration was detected in tumor tissue. *p < 0.05.

To investigate the role of HNRNPA2B1 on the NSCLC tumor growth, the in vivo assay using C57BL/6 mice was performed (Fig. 7A). The in vivo mice assay found that HNRNPA2B1 silencing repressed the tumor growth (Fig. 7B, C). The in vivo IFN-γ level analysis revealed that HNRNPA2B1 silencing up-regulated the IFN-γ level (Fig. 7D). The immumohistochemical (IHC) staining revealed that HNRNPA2B1 silencing reduced the LDHA positive cells, and promoted the CD8 positive cells (Fig. 7E, F). The in *vivo* analysis revealed that HNRNPA2B1 silencing up-regulated the IFN-γ level and Fe^2+^ level (Fig. 7G). Overall, these data indicated that HNRNPA2B1 silencing reduced the tumor growth, and accelerated the IFN-γ and Fe^2+^ level in vivo.

## Discussion

Tumor microenvironment refers to the internal and external environment of tumor cells, which is closely related to the occurrence, growth and metastasis of tumors. In the past two decades, a series of studies have revealed the critical role of the tumor microenvironment in regulating tumor progression [17, 18]. CD8^+^ T cells, also known as cytotoxic T lymphocytes, are the most important immune cells responsible for killing tumor cells [19]. Studies have indicated that CD8^+^ T cells are essential for the immune system’s reaction against cancer, and that anti-tumor immunity is mostly dependent on their capacity to eliminate tumor cells directly [20, 21]. Tumors, however, frequently adapt defense mechanisms to avoid this cytotoxic response. One such strategy is the overexpression of PD-L1, which binds to PD-1 on T cells and exhausts T cells while compromising the immune response [22]. How to stimulate the ability of CD8^+^ T cells to kill tumor cells, or to relieve the immunosuppression of CD8^+^ T cells, is an important research direction of tumor immunotherapy.

Here, our research focused on the m^6^A modification, an important branch of epigenetic modification [23], and test the potential roles of m^6^A reader enzyme HNRNPA2B1 on NSCLC antitumor response and ferroptosis. These findings made clear that HNRNPA2B1 functioned as an oncogenic element and connected with poor prognosis. In NSCLC, Wang et al. (2024) reported that hnRNPA2B1 was found in multidrug-resistant cell lines and hnRNPA2B1 silencing effectively inhibited cell proliferation, enhanced the stemness properties and induced apoptosis to sensitize NSCLC cells to chemotherapy in vitro and in vivo [3]. Unlike this report, our finding focused on the tumor immune escape and antitumor activity of lymphocytes. Results showed that, in the CD8^+^ T cells co-culture, HNRNPA2B1 repressed the CD8^+^ T cells’ antitumor response and alleviated NSCLC cells’ ferroptosis triggered by co-cultured CD8^+^ T cells.

For the NSCLC tumor immune escape and immunotherapy, emerging evidences are proposing and reported. As regarding NSCLC immune escape, such studies involve a wide range of immune cells, including natural killer cells, CD8^+^/CD4^+^ T cells, macrophages, and so on. And, of course, immunosuppressive cells are also involved in the immune regulation, e.g. MDSC and regulatory T cells. For example, hsa_circ_0001479 inhibited the infiltration of CD8^+^ T cells in NSCLC to mediate immune escape and accelerated the development and metastasis of NSCLC [24]. Moreover, in the NSCLC microenvironment, Jiang et al. (2024) found that TRIM29 is associated with CD8^+^ immune cell infiltration and regulates the antitumor T-cell immunity through TRIM29/IGF2BP1/PD-L1 axis [25]. Thus, these researches on CD8^+^ T cells hold extremely high value.

The goal of recent research has been to find ways to stimulate CD8^+^ T lymphocytes in the tumor microenvironment. One treatment strategy is to boost the cytotoxic potential of CD8^+^ T lymphocytes by blocking inhibitory pathways such as PD-1/PD-L1 and CTLA-4. In this present research, our data found that the IFN-γ could inspire the ferroptosis of NSCLC cells, and the ferroptosis specific inhibitor Fer-1 could repress the traits. Coincidentally, in the co-culture system with CD8^+^ T, IFN-γ was secreted by CD8^+^ T cells, which mediately induced the iron accumulation or ferroptosis. Therefore, this finding brought an insight to increase the effectiveness of CD8^+^ T cells to induce antitumor response and stimulate ferroptosis of NSCLC cells.

**Fig. 8:**
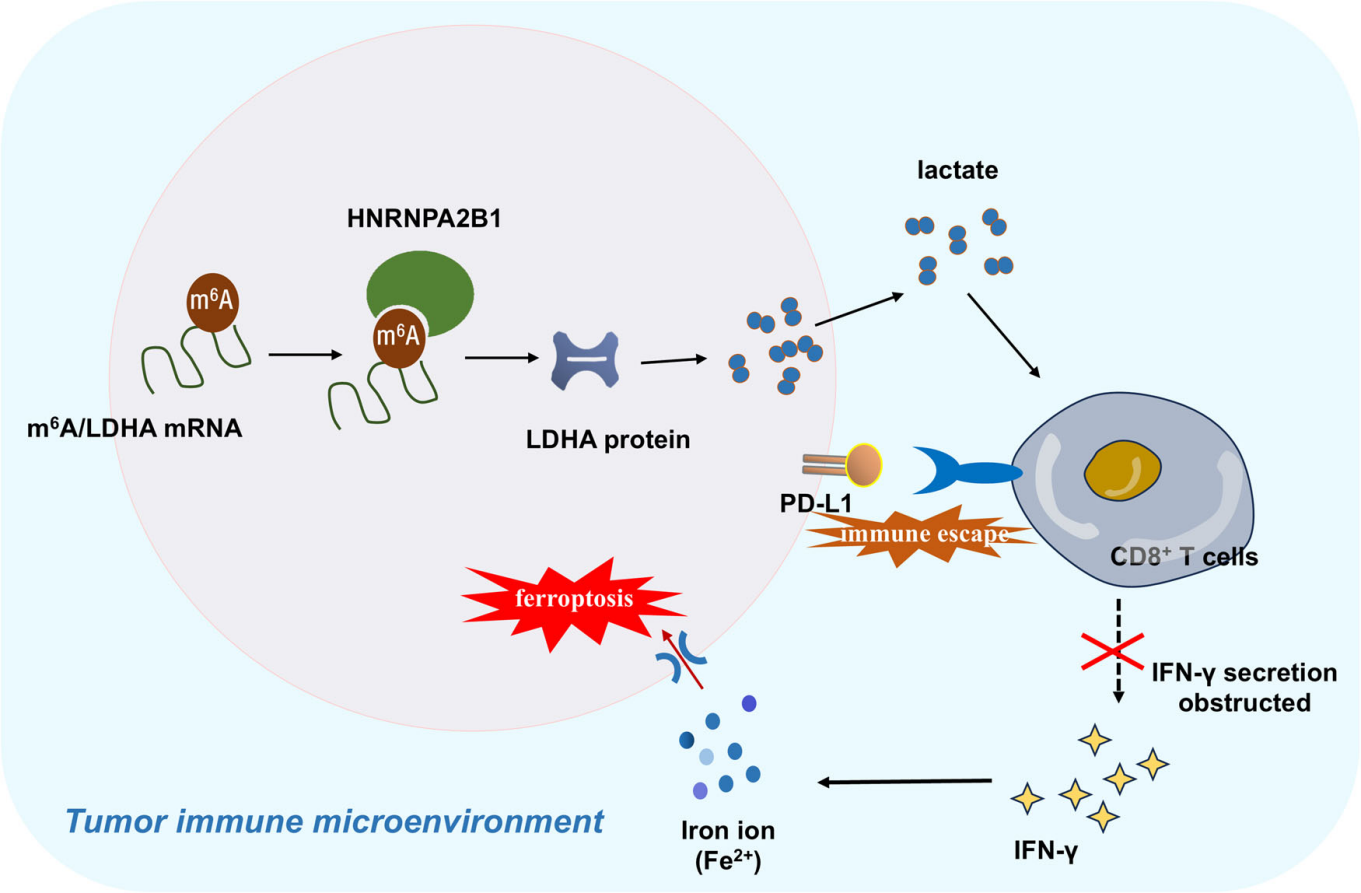
HNRNPA2B1 represses the ferroptosis of NSCLC cells to expedite immune escape. In brief, HNRNPA2B1 represses CD8^+^ T cells mediated IFN-γ secretion by inducing lactate to obstruct the iron uptake in NSCLC cells, thereby reducing the ferroptosis to accelerate NSCLC progression.

Overall, this present research constructs a signature based on m^6^A reader enzyme HNRNPA2B1 on NSCLC antitumor response and ferroptosis for adjuvanting anti-tumor immunotherapy. In NSCLC, elevated HNRNPA2B1 expression cells repressed the CD8^+^ T cells’ antitumor killing effect and further alleviated the ferroptosis (Fig. 8). HNRNPA2B1 represses the ferroptosis of NSCLC cells to accelerate immune escape. In brief, HNRNPA2B1 targeted LDHA/Lactate to repress CD8^+^ T cells mediated IFN-γ secretion to obstruct the iron uptake in NSCLC cells, thereby reducing the ferroptosis to accelerate NSCLC progression. This finding might help researchers to better explore the feasibility of tumor immunotherapy.

## Supplementary information

supplement Table S1

## Data Availability

The datasets generated during and/or analysed during the current study are available from the corresponding author on reasonable request.
