# Supplementary material for: HNRNPA2B1 confers immune escape of non-small cell lung cancer through targeting lactate/ferroptosis
Source: Cell Death Dis. 2025 Nov 21;17(1):49. doi: 10.1038/s41419-025-08232-5 (PMC12811398; doi:10.1038/s41419-025-08232-5)
Supplement: Supplementary file 1 — supplement Table S1 [file 41419_2025_8232_MOESM1_ESM.docx]

**supplementary table S1**. qRT-PCR primers sequences and shRNA sequences.

|  | Sequences |
| --- | --- |
| HNRNPA2B1 | F, 5’- ATTGATGGGAGAGTAGTTGAGCC-3’  R, 5’- AATTCCGCCAACAAACAGCTT-3’ |
| LDHA | F, 5’-ATGGCAACTCTAAAGGATCAGC -3’  R, 5’-CCAACCCCAACAACTGTAATCT-3’ |
| sh-HNRNPA2B1-1 | 5’- CAGAAATACCATACCATCAAT-3’ |
| sh-HNRNPA2B1-2 | 5’- GCTTCTTCCTATTTGCCATGG-3’ |
| GAPDH | F, 5’-CAGGAGGCATTGCTGATGAT-3’  R, 5’-GAAGGCTGGGGCTCATTT-3’ |
